# Supplementary material for: Analysis of Knowledge, Attitudes and Behaviours of Health Care Workers towards Vaccine-Preventable Diseases and Recommended Vaccinations: An Observational Study in a Teaching Hospital
Source: Vaccines (Basel). 2023 Jan 16;11(1):196. doi: 10.3390/vaccines11010196 (PMC9867139; doi:10.3390/vaccines11010196)
Supplement: Supplementary file 1 [file vaccines-11-00196-s001.zip › vaccines-2167453-supplementary.pdf]

Table S1. Results univariate analysis – percentages positive answers – Measles.

VARIABLE	GENDER		AGE				OCCUPATION				MARITAL STATUS (married)		EMPLOYER		CARE UNIT					YEARS OF SERVICE				YEARS OF SERVICE C.U.				CHILDREN	
(%)	F	M	20-29	30-39	40-49	>50	Phy	Nur	Resi	Other	No	Yes	Pub	Priv	Gen.	I.D.	Neon	Card.	Ped	>20	0-5	11-20	6-10	>20	0-5	11-20	6-10	Yes	No
**10_yes**	57.7	52.6	75	62.1	47.2	38.1	61.7	53.6	73.6	31	57.6	54.2	57	50	68.8	45.3	47.3	61.5	60	37.8	65.6	54.5	68.4	28.2	61.8	52.3	61.5	50	60.7
P	0.698		**<0.001**				0.14				0.18		0.709		0.052					**0.001**				**0.007**				0.101	
**12_agree**	16.9	24.2	17.1	12.6	24.5	25	6.4	22.3	11.3	34.5	17.5	21.4	18.1	26.3	21.5	21.3	10.8	26.9	20	25.3	15.6	18.2	18.4	17.9	16.6	27.3	26.9	19.2	19.1
P	0.284		0.373				**0.002**				0.212		**0.03**		0.198					**0.032**				0.44				0.105	
**13_agree**	85.9	76.8	88.2	84.2	77.4	83.3	97.9	78.8	96.2	69	84.7	82.4	84.8	76.3	80.6	81.3	85.1	80.8	95	81.6	85.2	87.3	78.9	87.2	83.9	84.1	76.9	82.3	84.8
p	0.052		0.578				**<0.001**				0.861		0.275		0.404					0.141				0.785				0.681	
**14_agree**	50.7	47.4	52.6	57.9	39.6	44	44.7	52.5	47.2	44.8	48.6	51.1	49.6	50	52.7	45.3	52.7	50	45	44.8	48.4	54.5	57.9	46.2	52.3	45.5	42.3	46.2	52.2
p	0.764		0.308				0.866				0.371		0.244		0.935					0.76				0.741				0.568	
**15_agree**	79.3	72.6	88.2	78.9	62.3	75	83	76	86.8	58.6	78	76.3	77	78.9	75.3	74.7	79.7	61.5	92.5	70.1	84.4	76.4	71.1	69.2	79.4	77.3	73.1	73.8	79.8
p	0.252		**<0.001**				**0.033**				0.726		0.742		0.063					**<0.001**				**0.001**				**0.007**	
**16_agree**	94.8	97.9	100	95.8	96.2	91.7	97.9	95.2	100	86.2	96	95.4	96.3	92.1	95.7	96	95.9	92.3	97.5	96.6	98.4	89.1	94.7	94.9	97.5	93.2	88.5	95.4	96.1
p	0.377		0.222				**0.024**				0.846		0.186		0.442					0.052				**0.019**				0.684	
**17_agree**	70.4	80	86.8	73.7	79.2	57.1	83	67.6	86.8	69	72.9	97	72.6	78.9	79.6	65.3	70.3	61.5	87.5	59.8	82.8	69.1	78.9	53.8	78.9	65.9	73.1	66.2	78.7
p	0.191		**<0.001**				**0.013**				0.963		**0.007**		0.107					**0.006**				**0.033**				**0.025**	
**18_agree**	92	86.3	97.4	93.7	84.9	83.3	95.7	87.7	100	79.3	91.5	88.5	90.4	89.5	89.2	82.7	97.3	80.8	100	81.6	95.3	89.1	94.7	79.5	92.5	86.4	96.2	86.2	93.3
p	0.284		**0.014**				**0.014**				0.639		0.36		**0.005**					**0.023**				**0.024**				**0.013**	
**19_agree**	92.5	96.8	98.7	94.7	98.1	85.7	93.6	92.7	100	98.7	93.2	94.7	93.7	94.7	92.5	92	97.3	84.6	100	89.7	95.3	96.4	94.7	87.2	95	93.2	96.2	93.1	94.4
p	0.332		**0.02**				0.185				0.862		0.685		0.062					0.508				0.404				0.717	
**20_agree**	77.9	73.7	86.8	77.9	77.4	65.5	78.7	76	79.2	72.4	76.8	76.3	75.2	86.8	78.5	72	79.7	61.5	85	69	82.8	72.7	78.9	71.8	79.9	75	61.5	76.9	76.4
p	0.201		0.059				0.138				0.766		0.099		0.241					0.34				0.382				0.994	
**21_yes**	16.9	21.1	36.8	22.1	9.4	2.4	8.5	15.6	37.7	13.8	22	13	17.8	21.1	28	18.7	10.8	0	20	4.6	28.9	1.8	36.8	7.7	23.6	4.5	15.4	7.7	25.8
p	0.471		**<0.001**				**0.002**				**0.034**		0.517		**0.002**					**<0.001**				**0.001**				**<0.001**	
**25_yes**	53.5	54.7	19.7	45.3	77.4	79.8	72.3	58.1	22.6	55.2	47.5	62.6	54.8	47.4	49.5	61.3	59.5	57.7	37.5	83.9	34.4	58.2	44.7	82.1	79.5	69.2	53.9	66.9	44.4
p	0.135		**<0.001**				**<0.001**				**0.021**		0.328		0.202					**<0.001**				**<0.001**				**<0.001**	
**26_agree**	83.6	81.1	93.4	89.5	77.4	69	85.1	79.3	94.3	79.3	83.6	81.7	82.2	86.8	84.9	81.3	87.8	57.7	87.5	67.8	89.8	83.6	92.1	74.4	87.4	75	73.1	77.7	86.5
p	0.565		**<0.001**				0.252				0.763		0.573		**0.043**					**<0.001**				**0.012**				0.108	

Table S2. Results univariate analysis – percentages positive answers – Mumps.

VARIABLE (%)	GENDER		AGE				OCCUPATION				MARITAL STATUS (married)		EMPLOYER		CARE UNIT					YEARS OF SERVICE				YEARS OF SERVICE C.U.				CHILDREN	
	**F**	**M**	**20-29**	**30-39**	**40-49**	**>50**	**Phy**	**Nur**	**Resi**	**Other**	**No**	**Yes**	**Pub**	**Priv**	**Gen**	**I.D.**	**Neon**	**Card**	**Ped**	**>20**	**0-5**	**11-20**	**6-10**	**>20**	**0-5**	**11-20**	**6-10**	**Yes**	**No**
**10_yes**	51.6	38.9	69.7	47.4	43.4	31	46.8	43	75.5	27.6	49.7	45	48.5	42.1	53.8	37.3	47.3	57.7	47.5	31	59.4	45.5	50	20.5	53.8	40.9	53.8	40	53.4
p	0.101		**<0.001**				**<0.001**				0.083		0.758		0.409					**0.003**				**0.015**				**0.035**	
**12_agree**	17.8	20	18.4	13.7	22.6	21.4	10.6	22.3	13.2	17.2	15.3	22.9	19.3	13.2	22.6	21.3	13.5	15.4	15	21.8	14.1	20	23.7	12.8	17.1	27.3	23.1	18.5	18.5
p	0.06		0.761				**0.004**				0.197		0.155		0.124					0.12				0.335				0.719	
**13_agree**	86.4	74.7	85.5	82.1	84.9	79.8	93.6	78.2	96.2	69	84.2	80.9	83.7	76.3	78.5	80	85.1	80.8	95	81.6	82.8	89.1	76.3	84.6	83.4	81.8	76.9	80.8	84.3
p	**0.039**		0.97				**0.001**				0.474		0.22		0.114					0.479				0.922				0.565	
**14_agree**	45.2	46.3	42.1	53.7	43.4	40.5	44.7	47.5	41.5	41.4	42.9	48.9	45.6	44.7	43	44	48.6	50	45	43.7	41.4	52.7	52.6	41	47.2	43.2	42.3	44.6	46.1
p	0.964		0.464				0.952				0.217		0.207		0.875					0.565				0.855				0.965	
**15_agree**	66.7	60	76.3	63.2	52.8	63.1	66	63.1	77.4	48.3	62.1	67.1	63.7	71.1	52.7	64	71.6	57.7	85	58.6	70.3	63.6	60.5	53.8	67.3	63.6	61.5	63.8	65.2
p	0.446		**<0.001**				0.121				0.304		0.399		**0.008**					**<0.001**				**0.001**				**0.003**	
**16_agree**	94.4	94.7	98.7	94.7	98.1	88.1	93.6	95	98.1	86.2	96	92.4	94.8	92.1	93.5	93.3	95.9	92.3	97.5	94.3	96.9	89.1	94.7	89.7	96.5	93.2	88.5	93.8	94.9
p	0.59		0.073				0.139				0.285		0.372		0.294					0.404				0.084				0.908	
**17_agree**	68.5	82.1	86.8	72.6	75.5	58.3	80.9	67.6	83	72.4	71.8	74	71.9	78.9	78.5	69.3	66.2	57.7	87.5	59.8	82	67.3	78.9	56.4	78.4	61.4	73.1	65.4	78.1
p	**0.041**		**0.003**				0.19				0.536		0.108		**0.006**					**0.002**				**0.043**				**0.005**	
**18_agree**	90.1	87.4	96.1	92.6	83	83.3	97.9	84.9	100	82.8	89.8	88.5	88.9	92.1	88.2	80	97.3	80.8	100	80.5	94.5	90.9	89.5	82.1	92.5	79.5	92.3	86.2	91.6
p	0.761		**0.016**				**0.005**				0.92		0.219		**0.001**					**0.008**				**0.004**				0.05	
**19_agree**	92	94.7	98.7	92.6	98.1	84.5	87.2	92.7	100	89.7	92.7	93.1	92.6	94.7	90.3	92	97.3	80.8	100	88.5	93.8	96.4	94.7	87.2	93.5	93.2	96.2	91.5	93.8
p	0.262		**0.017**				0.169				0.979		0.602		0.076					0.518				0.603				0.669	
**20_agree**	76.5	71.6	88.2	78.9	73.6	59.5	70.2	75.4	79.2	72.4	75.7	74	73.3	86.8	79.6	68	75.7	65.1	85	63.2	84.4	69.1	78.9	61.5	80.9	68.2	61.5	74.6	75.3
p	0.174		**0.002**				0.105				0.921		0.047		0.181					0.26				0.062				0.908	
**21_yes**	16.4	21.1	38.2	21.1	7.5	2.4	8.5	15.6	37.7	10.3	22.6	11.5	17.8	18.4	28	18.7	10.8	0	17.5	4.6	28.9	1.8	34.2	7.7	23.1	4.5	15.4	7.7	25.3
p	0.303		**<0.001**				**0.001**				0.26		0.293		**0.002**					**<0.001**				**0.001**				**<0.001**	
**25_yes**	39.9	38	18.4	31.6	52.8	59.5	46.8	42.5	18.9	48.3	36.7	43.5	40	36.8	35.5	42.7	43.2	46.2	32.5	60.9	25.8	54.5	15.8	53.8	31.7	61.4	42.3	50	32
p	0.058		**<0.001**				**0.012**				0.434		0.922		0.4					**<0.001**				**0.006**				**0.004**	
**26_agree**	81.7	81.1	94.7	87.4	75.4	66.7	80.9	78.2	94.3	79.3	83.1	79.4	80.7	86.8	82.8	80	86.5	57.7	87.5	65.5	89.8	80	92.1	71.8	86.9	70.5	73.1	75.4	86
p	0.693		**<0.001**				0.181				0.711		0.582		0.057					**<0.001**				**0.006**				**0.032**	

Table S3. Results univariate analysis – percentages positive answers – Rubella.

VARIABLE (N%)	GENDER		AGE				OCCUPATION				MARITAL STATUS (married)		EMPLOYER		CARE UNIT					YEARS OF SERVICE				YEARS OF SERVICE C.U.				CHILDREN	
	**F**	**M**	**20-29**	**30-39**	**40-49**	**>50**	**Phy**	**Nur**	**Resi**	**Other**	**No**	**Yes**	**Pub**	**Priv**	**Gen**	**I.D.**	**Neon**	**Card**	**Ped**	**>20**	**0-5**	**11-20**	**6-10**	**>20**	**0-5**	**11-20**	**6-10**	**Yes**	**No**
**10_yes**	54.5	45.3	72.4	52.6	45.3	35.7	57.4	46.4	75.5	31	53.1	49.6	52.2	57.4	61.3	41.3	47.3	61.5	50	35.6	64.1	49.1	50	25.6	57.3	47.7	53.8	44.6	56.7
p	0.241			**<0.001**				**0.002**			**0.006**		0.826				0.258				**0.002**				**0.027**			**0.001**	
**12_agree**	14.6	24.2	18.4	9.5	22.6	22.6	8.5	21.2	7.5	27.6	14.7	21.4	17	21	17.2	21.3	10.8	19.2	22.5	21.8	14.1	16.4	21.1	12.8	15.6	25	26.9	17.7	17.4
p	0.116			0.081				**0.01**			0.307		0.397				0.074				0.151				0.509			0.714	
**13_agree**	85.9	75.8	85.5	82.1	83	81	93.6	78.2	96.2	69	83.1	82.4	83.7	76.3	78.5	81.3	83.8	80.8	95	81.6	83.6	87.3	76.3	84.6	83.4	84.1	73.1	81.5	83.7
p	0.083			0.95				**<0.001**			0.807		0.18				0.395				0.224				0.873			0.674	
**14_agree**	46.9	49.5	47.4	55.8	43.4	41.7	44.7	50.8	43.4	41.4	46.3	49.6	47.8	47.4	48.4	46.7	48.6	50	45	44.8	44.5	52.7	57.9	41	50.4	43.2	42.3	46.2	48.9
p	0.873			0.423				0.774			0.279		0.187				0.993				0.45				0.555			0.893	
**15_agree**	71.4	64.2	78.9	66.3	60.4	69	72.3	67.6	81.1	51.7	67.8	71	78.9	71.1	61.3	70.7	70.3	61.5	87.5	66.7	73.4	67.3	63.2	61.5	70.9	68.2	69.2	70	68.5
p	0.308			**<0.001**				0.12			0.5		0.607				0.051				**<0.001**				**<0.001**			**0.003**	
**16_agree**	95.3	96.8	100	96.8	96.2	90.5	97.9	96.1	98.1	86.2	96.6	94.7	96.3	92.1	95.7	94.7	97.3	92.3	97.5	95.4	97.7	90.9	97.4	92.3	97	95.5	92.3	95.4	96.1
p	0.75			0.074				**0.036**			0.501		0.186				0.71				0.129				0.536			0.84	
**17_agree**	70.4	76.8	89.5	75.8	73.6	52.4	76.6	68.2	84.9	69	72.9	71.8	71.1	81.6	80.6	65.3	66.2	61.5	85	55.2	85.2	69.1	73.7	46.2	80.4	61.4	69.2	64.6	78.1
p	0.474			**<0.001**				0.079			0.926		**0.009**				0.053				**<0.001**				**<0.001**			**0.011**	
**18_agree**	90.6	88.4	96.1	93.7	84.9	83.3	95.7	87.2	100	79.3	91.5	87.8	90	89.5	88.2	84	95.9	80.8	100	82.8	94.5	89.1	92.1	79.5	92.5	84.1	96.2	86.2	92.7
p	0.375			0.051				**0.011**			0.488		0.341				**0.004**				**0.018**				**0.047**			**0.029**	
**19_agree**	92.5	94.7	98.7	93.7	98.1	84.5	89.4	92.7	100	89.7	92.7	93.9	93	94.7	91.4	92	97.3	80.8	100	88.5	94.5	96.4	94.7	87.2	94	93.2	96.2	92.3	93.8
p	0.315			**0.011**				0.225			0.87		0.593				0.74				0.481				0.535			0.719	
**20_agree**	76.5	72.6	86.8	78.9	77.4	59.5	70.2	76	79.2	72.4	76.3	74	73.7	86.8	79.6	68	77	61.5	85	64.4	83.6	70.9	78.9	64.1	80.4	70.5	61.5	75.4	75.3
p	0.317			**0.004**				0.114			0.876		0.63				0.183				0.62				0.101			0.995	
**21_yes**	19.2	20	42.1	22.1	9.4	2.4	8.5	17.9	39.6	10.3	24.9	12.2	18.9	23.7	28	20	12.2	3.8	22.5	5.7	31.3	3.6	34.2	10.3	25.1	4.5	15.4	8.5	27.5
p	0.416			**<0.001**				**0.001**			**0.011**		0.29				**0.002**				**<0.001**				**<0.001**			**<0.001**	
**25_yes**	44.6	36.8	18.4	30.5	62.3	64.3	51.1	44.7	26.4	41.4	33.9	53.4	43.3	34.2	35.5	50.7	50	42.3	27.5	66.7	26.6	47.3	31.6	64.1	32.7	61.4	50	54.6	33.1
p	0.056			**<0.001**				0.111			**0.002**		0.453				0.169				**<0.001**				**0.001**			**<0.001**	
**26_agree**	84	84.2	94.7	90.5	81.1	69	85.1	81.6	94.3	79.3	84.7	83.2	83.7	86.8	87.1	84	87.8	57.7	87.5	70.1	91.4	83.6	92.1	74.4	89.4	75	73.1	77.7	88.8
p	0.858			**<0.001**				0.373			0.935		0.618				**0.032**				**<0.001**				**0.003**			**0.028**	

Table S4. Results univariate analysis – percentages positive answers – Chickenpox.

VARIABLE	GENDER		AGE				OCCUPATION				MARITAL STATUS (married)		EMPLOYER		CARE UNIT					YEARS OF SERVICE				YEARS OF SERVICE C.U.				CHILDREN	
(N%)	F	M	20-29	30-39	40-49	>50	Phy	Nur	Resi	Other	No	Yes	Pub	Priv	Gen	I.D.	Neon	Card	Ped	>20	0-5	11-20	6-10	>20	0-5	11-20	6-10	Yes	No
10_yes	55.9	43.2	72.4	55.8	47.2	32.1	46.8	49.7	75.5	31	54.8	48.1	53	44.7	60.2	38.7	50	53.8	60	34.5	60.9	49.1	65.8	23.1	57.3	45.5	65.4	43.1	58.4
p	0.116		**<0.001**				**0.001**				**0.001**		0.592		0.085					**0.001**				**0.003**				**0.009**	
12_agree	11.7	24.5	11.8	9.5	24.5	20.2	10.6	17.3	5.7	31	14.1	17.6	14.1	26.3	16.1	16	12.2	23.1	15	20.7	11.7	14.5	18.4	12.8	13.6	20.5	26.9	17.7	14
p	**0.008**		0.176				**0.027**				0.117		0.146		0.063					0.167				0.574				0.118	
13_agree	85	77.9	85.5	85.3	73.6	83.3	89.4	79.3	94.3	72.4	84.7	80.2	83.3	78.9	78.5	85.3	81.1	76.9	95	83.9	82.8	80	84.2	89.7	82.8	79.5	76.9	77.7	86.6
p	0.314		0.358				0.058				0.566		0.723		0.289					0.507				0.747				0.122	
14_agree	48.4	51.6	51.6	56.8	41.5	42.9	42.6	53.6	43.4	44.8	49.2	49.6	49.3	50	52.7	48	51.4	46.2	42.5	46	46.9	50.9	63.2	43.6	52.3	43.2	46.2	45.4	52.2
p	.815		0.436				0.541				0.384		0.188		0.911					0.54				0.703				0.464	
15_agree	80.8	74.7	89.5	80	71.7	72.6	80.9	79.3	86.4	58.6	77.4	80.9	78.9	78.9	77.4	77.3	79.7	61.5	95	72.4	85.2	76.4	76.3	64.1	81.4	79.5	80.8	75.4	81.5
p	0.262		**0.001**				0.05				0.577		0.872		**0.029**					**0.001**				**0.002**				**0.045**	
16_agree	90.1	94.7	93.4	95.8	88.7	87.9	95.7	90.5	96.2	82.8	90.4	93.1	91.9	89.5	90.3	92	93.2	88.5	92.5	88.5	93	90.9	94.7	89.7	94	88.6	80.8	91.5	91.6
p	0.393		0.377				0.146				0.365		0.568		0.716					0.487				**0.042**				0.748	
17_agree	76.8		90.8	72.6	71.7	54.8	78.7	66.5	86.8	69	72.3	71.8	71.1	78.9	78.5	65.3	66.2	53.8	92.5	58.6	84.4	60	78.9	51.3	78.4	61.4	73.1	60.8	80.3
p	0.439		**<0.001**				0.34				0.931		**0.002**		**0.009**					**<0.001**				**0.006**				**0.001**	
18_agree	91.1	86.3	97.4	91.6	84.9	83.3	95.7	86.6	100	79.3	90.4	88.5	89.6	89.5	88.2	82.7	95.9	80.8	100	82.8	95.3	87.3	89.5	79.5	92.5	84.1	92.3	85.4	92.7
p	0.39		**0.008**				0.12				0.462		0.399		**0.005**					**0.007**				**0.006**				**0.029**	
19_agree	91.5	96.8	98.7	93.7	98.1	84.5	89.4	92.7	100	89.7	92.1	94.7	93	94.7	92.5	90.7	95.9	84.6	100	88.5	94.5	96.4	94.7	87.4	94.5	90.9	96.2	93.1	93.3
p	0.232		**0.008**				0.214				0.677		0.647		**0.045**					0.522				0.634				0.914	
20_agree	77.5	77.9	86.8	78.9	81.1	65.5	76.6	78.2	79.2	72.4	76.8	78.6	76.3	86.8	80.6	74.7	77	61.5	87.5	72.4	83.6	70.9	78.9	69.2	81.4	75	65.4	77.7	77.5
p	0.587		**0.027**				0.111				0.898		0.096		0.214					0.342				0.33				0.999	
21_yes	13.1	17.9	30.3	15.8	9.4	2.4	6.4	12.3	34	6.9	17.5	10.7	14.8	13.2	24.7	14.7	8.1	3.8	10	4.6	23.4	1.8	26.3	7.7	18.6	4.5	11.5	7.7	19.7
p	0.549		**<0.001**				**0.001**				**0.033**		0.934		**0.003**					**<0.001**				**0.018**				**<0.001**	
25_yes	81.2	75.8	78.9	80	83	77.4	87.2	77.1	81.1	79.3	79.7	79.4	79.6	78.9	80.6	78.7	78.4	76.9	82.5	82.8	81.3	72.7	76.3	82.1	79.4	79.5	76.9	76.9	81.5
p	0.521		0.551				0.375				0.942		0.715		0.714					**0.025**				0.986				0.545	
26_agree	82.2	81.1	90.8	86.3	81.1	69	85.1	77.7	94.3	79.3	82.5	80.9	81.5	84.2	80.6	82.7	89.2	57.7	85	70.1	87.5	80	92.1	76.9	85.9	72.7	73.1	76.2	86
p	0.517		**0.008**				0.085				0.648		0.712		0.134					**0.004**				0.083				0.059	

Table S5. Results univariate analysis – percentages positive answers – Pertussis.

VARIABLE (N%)	GENDER		AGE				OCCUPATION				MARITAL STATUS (married)		EMPLOYER		CARE UNIT					YEARS OF SERVICE				YEARS OF SERVICE C.U.				CHILDREN	
	**F**	**M**	**20-29**	**30-39**	**40-49**	**>50**	**Phy**	**Nur**	**Resi**	**Other**	**No**	**Yes**	**Publ**	**Priv**	**Gen**	**I.D.**	**Neo**	**Card**	**Ped**	**>20**	**0-5**	**11-20**	**6-10**	**>20**	**0-5**	**11-20**	**6-10**	**Yes**	**No**
**10_yes**	51.2	43.2	68.4	52.6	43.4	29.8	48.9	43.6	77.4	27.6	50.3	46.6	49.6	42.1	55.9	38.7	45.9	61.5	47.5	32.2	60.9	41.8	55.3	20.5	55.8	38.6	53.8	40	55.1
p	0.415			**<0.001**				**<0.001**			0.055		0.666				0.254				**0.002**				**0.002**			**0.01**	
**12_agree**	23.5	30.5	21.1	21.1	31.1	31	31.9	29.1	13.2	17.2	25.4	26	26.3	21.1	26.9	24	20.3	30.8	32.5	32.2	19.5	27.3	28.9	25.6	23.1	36.4	26.9	26.7	24.2
P	0.298			0.545				**0.002**			0.987		0.202				0.2				**0.031**				0.109			0.455	
**13_agree**	85.4	74.7	86.8	82.1	83	77.4	95.7	76.5	96.2	69	82.9	82.4	83	76.3	79.6	78.7	83.8	80.8	92.5	78.2	84.4	87.3	76.3	81.1	83.9	77.3	76.9	80	83.7
p	0.071			0.779				**<0.001**			0.886		0.356				0.352				0.213				0.826			0.652	
**14_agree**	43.7	45.3	39.5	52.6	43.4	39.3	44.7	45.8	39.6	41.4	41.2	48.1	44.1	44.7	41.9	45.3	45.9	50	40	42.5	39.8	50.9	52.6	41	45.7	38.6	46.2	44.6	43.8
p	0.959			0.485				0.919			0.099		0.178				0.939				0.408				0.734			0.941	
**15_agree**	68.5	66.3	75	68.4	60.4	65.5	74.5	64.8	81.1	51.7	66.1	70.2	67.4	71.1	59.1	69.3	68.9	53.8	92.5	63.2	73.4	69.1	57.9	56.4	70.9	70.5	57.7	67.2	69.1
p	0.718			**0.006**				0.134			0.591		0.312				**0.008**				**<0.001**				**0.002**			**0.018**	
**16_agree**	93	91.6	94.7	93.7	98.1	85.7	91.5	93.9	94.3	82.8	92.1	93.1	93.3	86.8	89.2	93.3	95.9	92.3	92.5	92	93	90.9	94.7	87.2	94	93.2	88.5	93.8	91.6
p	0.877			0.055				0.095			0.584		0.09				0.717				0.742				0.617			0.567	
**17_agree**	69	78.9	86.8	69.5	75.5	59.5	89.4	64.8	81.1	72.4	69.5	75.6	71.1	78.9	76.3	68	68.9	57.7	85	60.9	82	65.5	73.7	61.5	77.9	59.1	65.4	65.4	77
p	0.168			**0.014**				**0.022**			0.486		**0.012**				0.052				**0.015**				0.094			**0.028**	
**18_agree**	90.1	88.4	96.1	92.6	86.8	82.1	95.7	86.6	98.1	82.8	89.3	90.1	89.3	92.1	87.1	82.7	97.3	80.8	100	81.6	94.5	89.1	92.1	79.5	93	79.5	96.2	85.4	92.7
p	0.894			0.052				**0.028**			0.716		0.198				**0.001**				**0.008**				**0.005**			**0.045**	
**19_agree**	92	95.8	98.7	94.7	98.1	83.3	89.4	92.7	100	89.7	92.7	93.9	93	94.7	92.5	92	95.9	80.8	100	87.4	95.3	96.4	94.7	84.6	94.5	93.2	96.2	92.3	93.8
p	0.384			**0.004**				0.225			0.87		0.593				0.132				0.164				0.179			0.719	
**20_agree**	77	73.7	85.5	77.9	79.2	63.1	76.6	75.4	79.2	72.4	75.7	76.3	74.4	86.8	76.3	70.7	79.7	61.5	87.5	70.1	82	69.1	78.9	69.2	80.4	70.5	61.5	75.4	76.4
p	0.164			0.053				0.143			0.698		0.076				0.209				0.343				0.295			0.847	
**21_yes**	15.5	18.9	35.5	20	5.7	2.4	14.9	11.2	41.5	6.9	18.6	13.7	16.7	15.8	23.7	21.3	10.8	0	12.5	3.4	29.7	1.8	23.7	5.1	22.6	4.5	7.7	9.2	21.9
P	0.695			**<0.001**				**<0.001**			**0.028**		0.262				**0.001**				**<0.001**				**<0.001**			**<0.001**	
**25_yes**	24.4	16.8	5.3	22.1	34	29.8	27.7	20.1	13.2	41.4	22	22.1	21.5	26.3	20.4	24	23	23.1	20	26.4	16.4	30.9	18.4	25.6	19.1	27.3	30.8	28.5	17.4
p	**0.009**			**0.001**				**0.03**			0.396		0.552				0.842				0.115				**0.019**			0.076	
**26_agree**	82.6	81.1	93.4	87.4	79.2	67.9	83	79.3	92.5	79.3	83.1	80.9	81.5	86.8	81.7	86.7	81.5	57.7	85	77.8	89.8	80	92.1	71.8	87.9	70.5	73.1	75.4	87.1
p	0.647			**0.001**				0.312			0.85		0.542				**0.037**				**<0.001**				**0.014**			**0.029**	

Table S6. Results univariate analysis – percentages positive answers – Meningococcal disease.

VARIABLE (N%)	GENDER		AGE				OCCUPATION				MARITAL STATUS (married)		EMPLOYER		CARE UNIT					YEARS OF SERVICE				YEARS OF SERVICE C.U.				CHILDREN	
	**F**	**M**	**20-29**	**30-39**	**40-49**	**>50**	**Phy**	**Nur**	**Resi**	**Other**	**No**	**Yes**	**Pub**	**Priv**	**Gen**	**I.D.**	**Neon**	**Card**	**Ped**	**>20**	**0-5**	**11-20**	**6-10**	**>20**	**0-5**	**11-20**	**6-10**	**Yes**	**No**
**10_yes**	60.6	48.4	67.1	64.2	47.2	45.2	66	55.9	66	31	57.6	55.7	58.1	47.4	61.3	48	51.4	73.1	62.5	44.8	61.7	51.7	73.7	46.1	62.3	40.9	57.7	51.5	60.7
p	0.139		**0.002**				**0.021**				0.245		0.433		0.124					**0.001**				0.151				0.078	
**12_agree**	7.5	16.8	6.6	5.3	15.1	16.7	8.5	12.8	0	17.2	9	12.2	9.3	18.4	6.5	12	8.1	15.4	17.5	14.9	4.7	16.4	10.5	10.3	9	15.9	11.5	15.4	6.7
p	**0.01**		0.11				**<0.001**				0.55		**0.006**		0.313					0.086				0.819				**0.012**	
**13_agree**	89.2	78.9	86.8	86.3	84.9	85.7	97.9	81.1	96.2	72.4	84.7	87.8	87	78.9	80.6	85.3	89.2	84.6	95	87.4	85.2	89.1	81.6	94.9	85.9	81.8	80.8	84.6	87.1
p	0.056		0.866				**<0.001**				0.66		**0.007**		0.471					0.839				0.563				0.739	
**14_agree**	46.9	51.6	46.1	57.9	45.3	41.7	46.8	50.8	45.3	41.4	46.9	50.4	48.5	47.4	48.4	46.7	52.7	50	42.5	43.7	45.3	54.5	60.5	38.5	52.3	45.5	46.2	46.9	49.4
p	0.628		0.129				0.894				0.305		0.297		0.819					0.592				0.668				0.792	
**15_agree**	80.8	78.9	81.6	83.2	77.4	77.4	87.2	82.1	83	51.7	78.5	82.4	81.5	71.1	77.4	78.7	83.8	65.4	92.5	78.2	81.3	81.8	78.9	69.2	81.4	84.1	80.8	80.8	79.8
P	0.844		0.066				**0.005**				0.665		**0.045**		0.131					**0.02**				**0.004**				0.138	
**16_agree**	89.7	93.7	90.8	93.7	92.5	86.9	97.9	89.4	98.1	75.9	91	90.8	92.2	81.6	87.1	90.7	91.9	90.3	97.5	90.8	91.4	89.1	92.1	89.7	92	88.6	88.5	90	91.6
P	0.319		0.168				**0.006**				0.373		**0.026**		0.053					0.988				0.47				0.057	
**17_agree**	85.9	89.5	94.7	87.4	92.5	76.2	93.6	84.9	90.6	82.8	85.9	88.5	86.3	92.1	90.3	78.7	89.2	73.1	100	79.3	90.6	87.3	92.1	74.4	90.5	84.1	84.6	83.8	89.3
P	0.64		**0.012**				0.377				0.676		0.27		**<0.001**					0.099				0.74				0.092	
**18_agree**	94.4	92.6	97.4	96.8	94.3	86.9	97.9	92.7	100	82.8	93.8	93.9	94.1	92.1	92.5	89.3	100	84.6	100	87.4	95.3	98.2	97.4	82.1	96	93.2	96.2	91.5	95.5
P	0.124		**0.031**				**0.002**				0.437		**0.044**		**<0.001**					**0.005**				**0.001**				0.17	
**19_agree**	93	94.7	96.1	94.7	100	85.7	89.4	93.9	98.1	89.7	93.2	93.9	93.3	94.7	93.5	90.7	05.9	88.5	97.5	89.7	93.8	96.4	97.4	87.2	94.5	93.2	96.2	93.1	93.8
P	0.631		**0.04**				0.299				0.955		0.576		0.332					0.517				0.451				0.698	
**20_agree**	83.1	77.9	89.5	80	86.8	72.6	83	82.7	81.1	72.4	81.4	81.7	80.7	86.8	82.8	76	85.1	65.4	92.5	80.5	85.2	72.7	84.2	82.1	83.4	79.5	69.2	80.8	82
P	0.257		**0.042**				**0.027**				0.783		0.153		0.185					0.382				0.585				0.902	
**21_yes**	23.5	30.5	50	26.3	13.2	10.7	25.5	21.2	50.9	6.9	29.9	19.8	25.9	23.7	25.8	28	27	7.7	30	11.5	37.5	12.7	36.8	10.3	30.2	22.7	19.2	14.6	33.7
P	0.18		**<0.001**				**<0.001**				**0.027**		0.161		**0.027**					**<0.001**				0.406				**<0.001**	
**25_yes**	0.9	4.2	1.3	1.1	0	4.8	2.1	1.7	0	6.9	1.7	2.3	1.5	5.3	2.2	1.3	4.1	0	0	3.4	7.8	1.8	0	2.6	1	4.5	3.8	2.3	1.7
p	0.142		0.208				**0.014**				0.252		**<0.001**		0.723					0.636				0.611				0.514	
**26_agree**	86.4	83.2	97.4	89.5	79.2	73.8	89.4	83.8	94.3	72.4	85.3	85.5	85.9	81.6	88.2	84	86.5	61.5	95	71.3	91.4	87.3	94.7	69.2	89.4	84.1	80.8	80.8	88.8
p	0.358		**<0.001**				0.383				0.64		0.773		**0.03**					**0.001**				**0.009**				0.146	
